# Exploring the Hemolymph of the Pill Millipede *Arthrosphaera lutescens* (Butler, 1872): Chemical Composition, Bioactive Properties, and Computational Studies

**DOI:** 10.3390/cimb47060434

**Published:** 2025-06-09

**Authors:** Priyanka Palakkaparambil, Veena Venugopal, Gouthami Vijayan, Mohammed Amjed Alsaegh, Varun Thachan Kundil, Arun Kumar Gangadharan, Ovungal Sabira, Aswathi, A. V. Raghu, Kodangattil Narayanan Jayaraj, Anthyalam Parambil Ajaykumar

**Affiliations:** 1 Division of Biomaterial Sciences, Department of Zoology, Sree Neelakanta Government Sanskrit College (Affiliated to University of Calicut), Pattambi, Palakkad 679306, Kerala, India; priyapkd1995@gmail.com (P.P.); veenarun86@gmail.com (V.V.); sabiraovungal@gmail.com (O.S.); achuthy7@gmail.com (A.); 2 Department of Botany, Sree Neelakanta Government Sanskrit College, Pattambi, Palakkad 679306, Kerala, India; drgouthamiv@gmail.com; 3 Department of Oral and Craniofacial Health Sciences, College of Dental Medicine, University of Sharjah, Sharjah P.O. Box 27272, United Arab Emirates; malsaegh@sharjah.ac.ae; 4 Department of Biotechnology and Microbiology, Kannur University, Kannur 670661, Kerala, India; varuntk.me2@gmail.com; 5 Department of Molecular Biology, Kannur University, Kannur 670661, Kerala, India; arunkumar@kannuruniv.ac.in; 6 Kerala Forest Research Institute, Peechi, Thrissur 680653, Kerala, India; avraghu@kfri.res.in; 7 Basic Medical and Dental Sciences Department, Centre for Medical and Bio-Allied Health Sciences Research, Ajman University, Ajman P.O. Box 346, United Arab Emirates

**Keywords:** pill millipede, hemolymph, metabolites, bioactive properties, molecular docking

## Abstract

Most studies on the *Arthrosphaera* genus, or giant pill millipedes, focus on its taxonomy, distribution, and ecology. Therefore, this investigation aimed to explore the chemical composition and bioactive properties of the hemolymph of the giant pill millipede Arthrosphaera lutescens (Butler, 1872). Chemical characterization of hemolymph was performed using gas chromatography–mass spectrometry (GC-MS) and liquid chromatography–quadrupole time-of-flight mass spectrometry (LC-MS Q-TOF), revealing a complex array of over 200 compounds. The bioactive properties of hemolymph were determined by using radical scavenging capacity (DPPH assay); antibacterial activity against human pathogens like Escherichia coli (Migula, 1895) Castellani and Chalmers 1919, *Klebsiella pneumonia* (Schroeter, 1886) Trevisan 1887, and Staphylococcus aureus (Rosenbach, 1884); and cytotoxicity against Dalton’s lymphoma ascites (DLA) cells using the trypan blue assay. The hemolymph showed radical scavenging properties and antibacterial and cytotoxic activity. Among the identified metabolites, 1,2-dimethoxy-13-methyl-[1,3]benzodioxolo[5,6-c]phenanthridine (DMBP) emerged as a promising candidate due to its high abundance and bioactivity profile, showcasing therapeutic potential against both lymphoma and *S. aureus* in further docking studies. Computational analysis identified key T-cell lymphoma targets, with molecular docking suggesting DMBP’s anticancer properties through interactions with proteins like AKT1 and mTOR. Additionally, docking revealed DMBP’s antibacterial effects via interactions with proteins such as Sortase-A and DNA gyrase. This research underscores the potential pharmaceutical applications of metabolites from giant pill millipedes.

## 1. Introduction

Among terrestrial arthropods, diplopods (millipedes) are the third-largest group, with 12,000 described species [1]. They are also considered major saprophagous organisms that play a significant role in enhancing soil fertility. Sphaerotheriida, or giant pill millipedes, is one of the diverse orders present in diplopods and contains five families with 351 described species [2]. They are widely recognized as a monophyletic order within the class Diplopoda, subclass Pentazonia [3], with origins presumably tracing back to the Paleozoic era [4]. Within the subclass Pentazonia, Sphaerotheriida are the largest members. Pill millipedes are among the most captivating groups of diplopods, known for their remarkable size, color, and natural ability to roll into a ball (or pill) as a defense mechanism. Sphaerotheriida is one of the most interesting millipede orders because of its large size (10–100 mm) and the presence of stridulating organs in both males and females [5].

In peninsular India, 27 species of pill millipedes belong to the *Arthrosphaera* genus [6]. The genus *Arthrosphaera* is regarded as endemic to the forests of the Western Ghats and Sri Lanka. They are most active during the monsoon and post-monsoon seasons, and their presence has been noted in both low- and high-altitude areas [7]. *A. lutescens* (Butler, 1872) is one of the most fascinating species among them. *A. lutescens* are vital contributors to soil health and fertility and play an essential ecological role in the Western Ghats, one of the world’s most critical biodiversity hotspots. Like earthworms, these millipedes facilitate nutrient cycling by breaking down organic material and enriching soil quality, thereby supporting forest ecosystem sustainability [8]. The characteristic morphological features of *A. lutescens* are as follows: their tergites are olivaceous in color, a thin band of orange-brown or a band of full brown at the posterior margin is present, the head is dark brown, the antennae have a dark-green color, the legs are greenish, and the anterior part of the thoracic shield is black, while the posterior zone is green. They are usually found in the region of areca plantations and semi-evergreen to evergreen forests [7]. Most of the literature on these giant pill millipedes is interested in their distribution, abundance, and ecology. They are one of the least studied groups among diplopods [5].

Despite their ecological significance, research on the biochemical properties and hemolymph of pill millipedes remains limited, especially within the unique environmental context of the Western Ghats. In millipedes, hemolymph serves as the primary extracellular fluid, playing a vital role in the transport of nutrients, waste products, and hormones between tissues and cells. It is considered a static reservoir of metabolites, and any changes in the physiological state of the insect affect the dynamic nature of the hemolymph [9]. GC-MS and LC-MS analyses are valuable tools for studying the composition of millipede hemolymph, enabling the identification and quantification of various metabolites such as sugars, amino acids, lipids, and hormones. These techniques provide important insights into the physiological condition, health status, and environmental stress responses of millipedes [10]. In the knowledge so far, the study of the hemolymph of millipedes involves its osmoregulation [11], composition related to seasonal change [12], amino acid composition [13], and organic constituents. Physio-chemical properties like protein content, pH, and osmolarity present in the hemolymph of chilopods and diplopods were also explored [14]. The analysis of the chemical composition of arthropod hemolymph is significant for understanding compensatory mechanisms involved in stress-associated changes [15].

There is growing interest in the pharmacological potential of these arthropod-derived compounds, as they may possess biochemical properties such as antimicrobial, antioxidant, and cytotoxic activities. For example, the hemolymph from scarab beetles has been found to have antibacterial activity against both Gram-positive and Gram-negative bacteria such as *Escherichia coli* (Migula, 1895) Castellani and Chalmers 1919, *Enterobacter cloacae* (Jordan, 1890) Hormaeche and Edwards 1960, *Staphylococcus* (Rosenbach, 1884), etc. [16]. Studies have revealed that many alkaloids from this possess cytotoxicity against cancer cells due to their ability to disrupt cellular DNA and protein synthesis, which can be useful in inhibiting tumor growth [17]. Similarly, antioxidant compounds, including flavonoids and terpenoids, neutralize free radicals, potentially offering protection against oxidative stress and associated diseases [18]. Given the wide variety of bioactive compounds in these hemolymphs, analyzing their biochemical composition may reveal novel therapeutic agents or agricultural applications. This study investigates the chemical composition and bioactive properties of the hemolymph of *A. lutescens* (Butler, 1872), a pill millipede species endemic to the Western Ghats, using gas chromatography–mass spectrometry (GC-MS) and liquid chromatography–quadrupole time-of-flight mass spectrometry (LC-MS Q-TOF). These methods allow for an extensive profiling of both volatile and non-volatile biochemicals such as terpenoids, sulfur compounds, glycosides, and alkaloids. To explore the potential biotechnological and pharmacological uses of these compounds, various bioactivity tests are conducted. These include antioxidant assessments, antibacterial activity tests against common pathogens, and cytotoxicity tests on cancer cell lines. Additionally, the study incorporates computational analyses to predict the biochemical properties of the hemolymph. This study aims to enhance the understanding of the role of pill millipede hemolymph in chemical ecology and evaluate its potential therapeutic applications. Through this approach, the research contributes to the broader knowledge base regarding the utilization of invertebrate-derived substances in scientific applications.

## 2. Materials and Methods

### 2.1. Collection of A. lutescens and Preparation of Hemolymph

**Figure 1:**
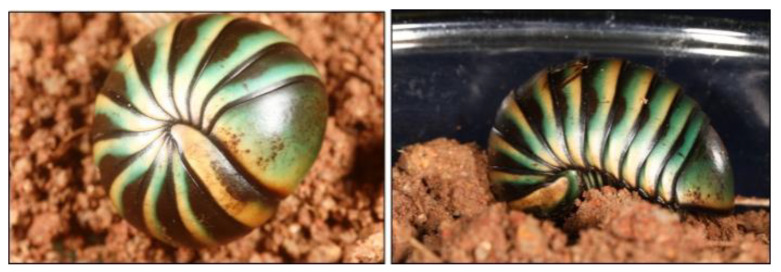
Pill millipede Arthrosphaera lutescens.

*Arthrosphaera lutescens* (Butler, 1872) (Figure 1) were collected from the forest areas of the Western Ghats, Seetharkundu, (10°33′18″ N 76°42′55″ E 1077 m) Kollengode, Palakkad, Kerala, India, which is recognized as a global biodiversity hotspot. The collection was conducted during the monsoon and post-monsoon seasons. Samples were collected according to ethical guidelines, ensuring minimal disturbance to the natural habitat. Hemolymph was obtained by gently stimulating the ventral side of the pill millipede; it was collected using sterile microcapillary tubes and immediately transferred into pre-labeled, amber-colored vials to protect against light degradation. Samples were stored at −20 °C to preserve the chemical integrity until further analysis.

### 2.2. Chemical Extraction of Hemolymph

Approximately 1 mL of the hemolymph was diluted in methanol (HPLC grade) to extract the compounds effectively. This was vortexed for 2 min to maximize extraction efficiency. The solution was then centrifuged at 1956.5× *g* for 5 min at 4 °C to remove any particulate matter. The filtered extract was stored at −20 °C and used for both GC-MS and LC-MS analysis.

### 2.3. Gas Chromatography–Mass Spectrometry (GC-MS) Analysis

The GC-MS analysis of the hemolymph of the pill millipede was conducted using a Shimadzu Nexis GC-2030 system equipped (Shimadzu corporation, Kyoto, Japan) with an AOC-30/20i autosampler and an SH-I-5Sil MS column (30.0 m length, 0.25 mm inner diameter, and 0.25 µm film thickness). The GC-MS solutions software (GCMS-QP2020 Series) was used for data acquisition, and the NIST 20 library was utilized for compound identification. The oven temperature was initially set to 40 °C for 1 min, followed by an increase of 10 °C/min to 290 °C, which was maintained for 5 min. The injection temperature was 260 °C, with splitless injection mode and a sample volume of 1 µL. Helium was used as the carrier gas in linear velocity mode with a total flow of 24.9 mL/min, a column flow of 1.04 mL/min, and a purge flow of 3.0 mL/min. The mass spectrometer operated with an ion source temperature of 200 °C and an interface temperature of 290 °C, scanning in the *m*/*z* range of 35–500 with a solvent cut time of 6.50 min. The compounds were identified based on mass spectra comparison with the NIST 20 library.

### 2.4. Liquid Chromatography–Quadrupole Time-of-Flight Mass Spectrometry (LC-MS Q-TOF) Analysis

LC-MS Q-TOF analysis was conducted on Agilent technologies. Chromatographic separation was achieved using a C18 column (100 × 2.1 mm^2^, 1.8 μm particle size) maintained at 25 °C. The mobile phase consisted of solvent A (water with 0.1% formic acid) and solvent B (acetonitrile with 0.1% formic acid), with a gradient program as follows: starting with 95% A and 5% B, increasing to 100% B over 25 min, then holding at 100% B for 5 min, and returning to the initial conditions over 5 min. The flow rate was maintained at 0.3 mL/min, and the injection volume was set to 5 μL. The mass spectrometer was operated in positive ion mode over a mass range of *m*/*z* 50–1200. Instrument parameters included a capillary voltage of 3500 V, a drying gas temperature of 325 °C, a drying gas flow rate of 8 L/min, and a nebulizer pressure of 35 psi. Data processing was performed using Agilent MassHunter software (Version 12), which identified molecular features based on retention time, *m*/*z* values, and peak area. Compounds were identified by comparing their *m*/*z* values with those in the METLIN and HMDB libraries.

### 2.5. Antioxidant Activity Assessment (DPPH Assay)

The antioxidant capacity of the hemolymph was evaluated using the DPPH radical scavenging assay. A stock solution of 0.1 mM DPPH in methanol was prepared and stored in the dark. Varying concentrations of the hemolymph (20 µg/mL, 40 µg/mL, 60 µg/mL, 80 µg/mL, and 100 µg/mL) were mixed with the DPPH solution in 1:1 ratios and allowed to react for 30 min in the dark. Absorbance was measured at 517 nm using a UV–visible spectrophotometer. Ascorbic acid was used as positive control; radical scavenging activity was calculated using the following formula:

S% is calculated as [(A control − A sample)/A control] × 100. The IC50 value, or the concentration at which 50% scavenging activity is achieved, was determined for the extract.

### 2.6. Evaluation of Antibacterial Activity

Antibacterial activity was assessed against human-disease-causing pathogens like Escherichia coli (Migula, 1895) Castellani and Chalmers 1919, Staphylococcus aureus Rosenbach, 1884), and *Klebsiella pneumonia* (Schroeter, 1886) Trevisan 1887 using the agar disc diffusion assay method. The spore suspension of these bacteria was added to sterile Muller–Hinton medium before solidification. Then, it was poured into a sterile petri dish (9 cm in diameter) and spread using a sterile cotton swab. A volume of 20 µL of hemolymph was pipetted into sterile discs of 6 mm and placed at the center of the petri dish. The antibiotic tetracycline was used as a control [19]. The plates were incubated at 37 °C for 24 h, after which zones of inhibition were measured in millimeters. The diameter of the inhibition zone was used to determine the antibacterial efficacy of the secretion extract.

### 2.7. Cytotoxicity Evaluation (Trypan Blue Exclusion Assay)

The cytotoxicity of the hemolymph was tested on Dalton’s lymphoma ascites (DLA) cells. DLA cells were cultured in RPMI-1640 medium supplemented with 10% fetal bovine serum and antibiotics. The cells were incubated with varying concentrations of the hemolymph extract for 24 h. The control tubes contained only cell suspension. Following incubation, the viability of the cells was determined using the trypan blue exclusion method. Equal volumes of cell suspension and trypan blue dye were mixed, and viable (unstained) and non-viable (stained) cells were counted using a hemocytometer. Cell viability was calculated as follows:

Percentage cytotoxicity = number of dead cells/number of live cells + number of dead cells × 100.

The concentration at which 50% cell death occurred (IC50) was calculated from dose–response curves to assess cytotoxic potential.

#### Statistical Analysis and Data Representation

Cytotoxicity and antioxidant (DPPH) assays were each conducted in five independent trials. The results are expressed as mean values ± standard deviation (SD), expressing a measure of variability across replicates. To assess the statistical significance of differences between the experimental groups and the control, the non-parametric Mann–Whitney U test was carried out. For the cytotoxicity assay, the percentage of cytotoxicity was calculated, and a graphical representation of the data was plotted using Microsoft Excel. In the DPPH assay, the effective concentration required to achieve 50% scavenging activity (EC_50_) was determined from the dose–response curve using linear regression analysis and visualized using Origin data analysis software (Version 10.0). For the antibacterial assay, three independent trials were performed, and the results are reported as the average of these replicates.

### 2.8. Data Analysis and Compound Selection for Docking Studies

The GC-MS and LC-MS analyses of the hemolymph revealed the presence of over 200 compounds, including metabolites with documented antimicrobial, antioxidant, and cytotoxic properties. Among these, 1,2-dimethoxy-13-methyl-[1,3]benzodioxolo[5,6-c]phenanthridine was chosen for molecular docking studies due to its high abundance and broad bioactivity profile.

#### Computational Studies: Mode of Action of DMBP Against Lymphoma

Screening of potential targets in T-cell lymphoma: The data for potential targets of active compound, DMBP, were obtained from Swiss target prediction [20] by entering the canonical SMILES and selecting “Homo sapiens” as the species. In contrast, the T-cell-lymphoma-related targets were gathered from three reliable sources: Gene Card [21,22], DisGeNet [23], and the OMIM database [24]. The search was performed using the keyword “T cell lymphoma”. After downloading the relevant data, the targets from these databases were merged, with duplicate entries removed. To identify the overlapping targets between the compound and T-cell lymphoma, a Venn diagram was constructed using the bioinformatics tool developed by Ghent University, Belgium [25].

Prediction of protein–protein interaction network and hub genes: The STRING database [26] was used to investigate the protein–protein interactions (PPIs) of the common genes, and “Homo sapiens” was chosen as the organism. The confidence in the interaction among the target proteins was set to the highest confidence level, >0.9. Cytoscape (Version 3.10.3) [27] was used to visualize the generated PPI network. The Cyto Hubba plugin was used to highlight nodes with high degrees within the network to discover important hub genes [28]. The strongest connections within the network were highlighted by the most prominent genes with the highest level of interaction.

Gene ontology analysis: Gene ontology was performed by using Funrich software (Version 3.1.4) [29]. It employs gene ontology analysis to classify gene functions into biological processes, cellular components, and molecular functions. The cut-off method with probability scores below 0.05 was applied to select the top GO annotations.

Molecular docking: Three-dimensional structure of the most promising candidate proteins from gene ontology analysis; AKT1 kinase, HSP90AA1, MAPK1, mTOR, and MDM2 were downloaded from PDB database. The obtained structures were prepared using the protein preparation wizard of Schrodinger, where missing loops and side chains were fixed. All crystallographic water molecules were removed, and the structure was minimized using the OPLS_2005 force field [30]. The docking site was specified using the receptor grid generation module, where the center of the co-crystalized ligand was selected. In the case of proteins with no co-crystalized ligand, a site map module was used to predict the active sites, and the coordinates of the top site were used for grid generation. The structure of ligand,1,2-dimethoxy-13-methyl-[1,3]benzodioxolo[5,6-c]phenanthridine (DMBP) was downloaded from the PubChem database and prepared using the ligprep module of the Schrodinger suite. The glide module of Schrodinger was used for protein–ligand docking, and extra precision docking was conducted, keeping ligand sampling flexible [31]. The binding energy was calculated using the prime module’s MMGBSA method [32].

Docking studies against *S. aureus* drug targets: In vitro studies have demonstrated that DMBP exhibits antibacterial activity against *S. aureus*. To explore potential protein targets computationally, docking studies were conducted on five essential *S. aureus* proteins identified from the literature: sortase-A, DNA gyrase, dihydrofolate reductase (DHFR), dehydrosqualene synthase (CrtM), and phosphotransacetylase. These proteins play critical roles in bacterial survival and pathogenicity, including cell wall formation, DNA supercoiling, nucleotide synthesis, and oxidative stress protection. The 3D structures of the selected proteins were retrieved from the Protein Data Bank (PDB) with the following IDs: sortase-A (1T2P), DNA gyrase (3U2D), DHFR (2W9S), dehydrosqualene synthase (CrtM, 2ZCZ), and phosphotransacetylase (4E4R). Protein preparation was performed using the protein preparation wizard in Schrödinger, which involved adding missing loops and side chains, removing crystallographic water molecules, and minimizing the structures using the OPLS_2005 force field. The docking sites were defined using the receptor grid generation module. For proteins with co-crystallized ligands, the center of the ligand was selected as the docking site. For proteins without co-crystallized ligands, the grid was set based on active site residues. Docking was conducted using Schrödinger’s glide module with extra precision (XP) docking, and ligand sampling was set to flexible. Binding energy predictions were calculated using the MMGBSA method.

## 3. Results

### 3.1. GC-MS Analysis

**Figure 2:**
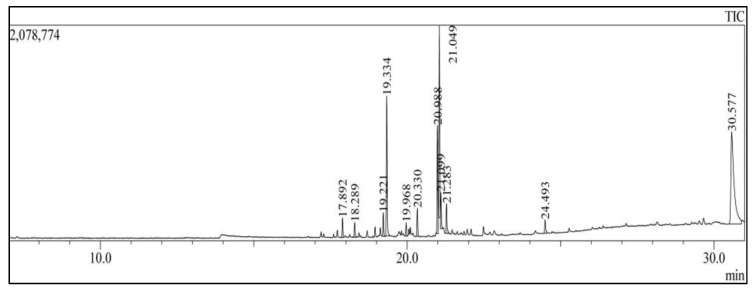
The base peak chromatogram of various compounds presents in the hemolymph of *A. lutescens* using GC-MS analysis.

The analysis using GC-MS of the hemolymph (Figure 2) from *A. lutescens* (Butler, 1872) revealed a variety of compounds (Table 1). The most abundant group of components from the analysis are fatty acids. Tridecanoic acid, pentadecanoic acid, hexadecanoic acid, heptadecanoic acids, and octadecanoic acids are observed in this group. Additionally, other noteworthy compounds comprised unsaturated fatty acid methyl esters, such as 9-hexadecenoic acid methyl esters and linoleic acid methyl esters. Most of the compounds like carboxylic acids are found in the hemolymph of arthropods. Table 2 shows the bioactivity of selected components; most of the components show anticancer, antibacterial, and antioxidant properties.

### 3.2. LC-MS Q-TOF Analysis of Hemolymph

**Figure 3:**
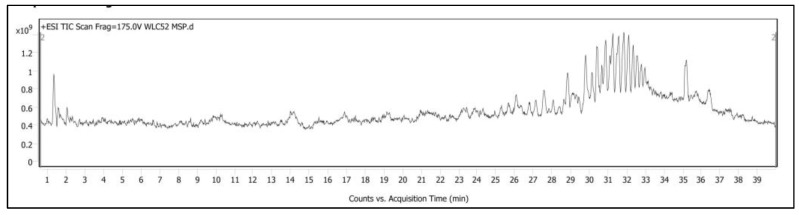
Total ion chromatogram (TIC) of LC-MS analysis of the hemolymph of the pill millipede.

The LC-MS analysis of the pill millipede’s hemolymph (Figure 3) uncovered a complex profile, identifying over 200 individual compounds.

The LC-MS analysis revealed a wide range of metabolites including sugars, amino acids (and their derivatives), fatty acids, esters, alkaloids, and phenolic compounds (Table 3). Among the sugars, trehalose, lactose, maltose, and others have identical retention times (1.529 min) and mass-to-charge ratios (m/z 342.1156). Amino acids and modified derivatives, including phenylalanine, valine, leucine, and more complex peptides like phenylalanyl-tyrosine and anserine, exhibited varied retention times and m/z values, reflecting differences in polarity and molecular weight. Fatty acids such as palmitic, lauric, and methyl myristic acid were eluted at higher retention times (30–38 min), consistent with their hydrophobic nature, and showed high identification scores. Esters identified, including butyl dodecanoate and methyl dodecanoate, demonstrated similar elution profiles and m/z values to their fatty acid counterparts, highlighting potential isobaric overlap. Alkaloids, including valerianine and phenanthridine derivatives, were detected with high confidence, suggesting bioactive potential. Phenolic compounds were observed at later retention times, with high scores, and included both substituted phenols and polycyclic structures like naphthalene dihydrodiol.

### 3.3. Antioxidant Activity of Pill Millipede Hemolymph Assessed by DPPH Assay

**Figure 4:**
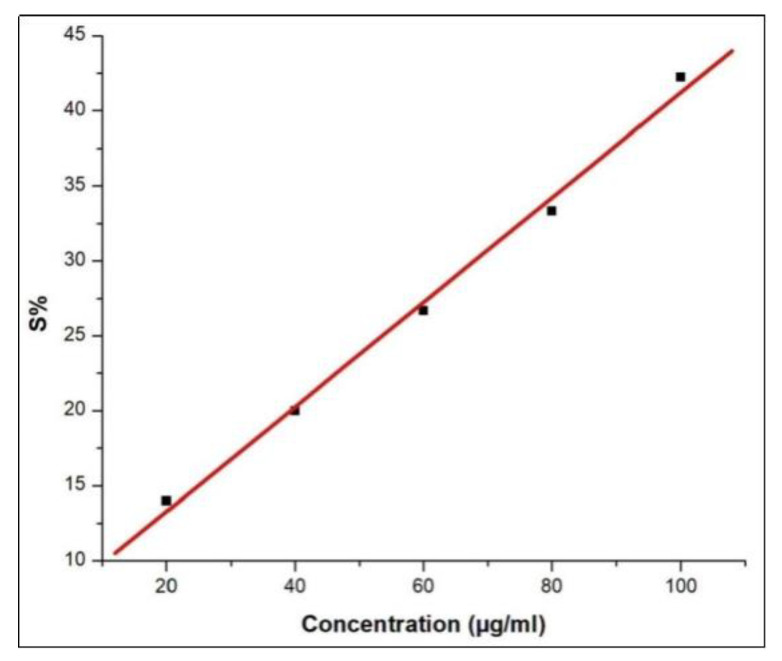
DPPH radical scavenging activity of the hemolymph of *A. lutescens*. The graph illustrates the percentage inhibition of DPPH radicals at various concentrations of hemolymph.

The antioxidant potential of *A. lutescens* hemolymph was evaluated using the DPPH (2,2-diphenyl-1-picrylhydrazyl) radical scavenging assay. Different concentrations of the hemolymph were tested to determine their effectiveness in neutralizing DPPH free radicals, thereby quantifying the free radical scavenging activity. As depicted in the trendline (Figure 4), a concentration-dependent increase in the DPPH radical scavenging activity of the hemolymph extract was observed. The graph illustrates a clear trend of decreasing DPPH radical concentration with increasing hemolymph concentration, suggesting a linear pattern. This trend supports the dose–responsive antioxidant potential of the hemolymph of pill millipedes across the tested concentrations.

### 3.4. Anticancer Activity of Pill Millipede Hemolymph Against DLA Cells

**Figure 5:**
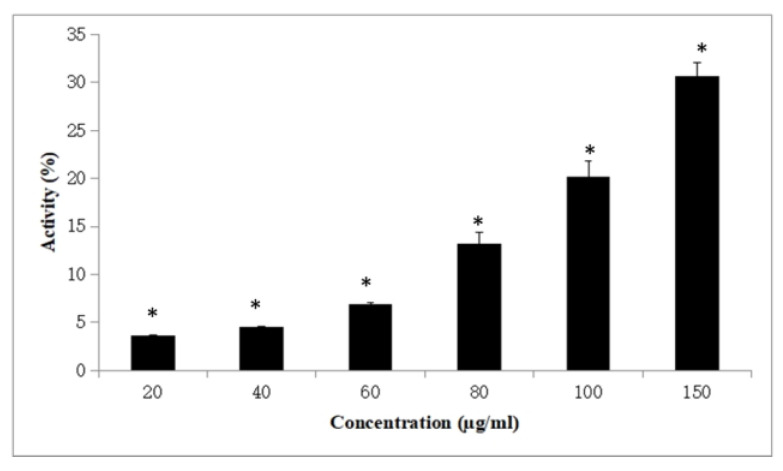
Anticancer activity of *A. lutescens* hemolymph against DLA cells. A significant (*) difference was found between the experimental and control groups (Mann–Whitney U = 33.0, *p* = 0.015). Data are presented as mean ± SD from five independent trials.

The anticancer potential of pill millipede hemolymph was investigated by examining its cytotoxic effects on Dalton’s lymphoma ascites (DLA) cells using the trypan blue exclusion method. Various concentrations of the hemolymph were tested to evaluate its dose-dependent impact on cell viability. As illustrated in the bar graph (Figure 5), a statistically significant difference was found between the experimental and control groups (Mann–Whitney U = 33.0, *p* = 0.015). Moreover, it indicates that the hemolymph exerts a dose-dependent cytotoxic effect. At the lowest concentration of 20 µg/mL, the cytotoxic effect was modest, approximately 4%. However, as the concentration increased to 40 µg/mL and 60 µg/mL, the activity exhibited slight increases to approximately 5% and 7%, respectively. A more significant enhancement in cytotoxicity was noted at higher concentrations. At 80 µg/mL, the hemolymph demonstrated approximately 13% cytotoxicity, which further escalated to around 20% at 100 µg/mL. The highest concentration tested, 150 µg/mL, revealed the most prominent effect, with approximately 31% of the DLA cells rendered non-viable following treatment.

### 3.5. Antibacterial Activity of Pill Millipede Hemolymph

**Figure 6:**
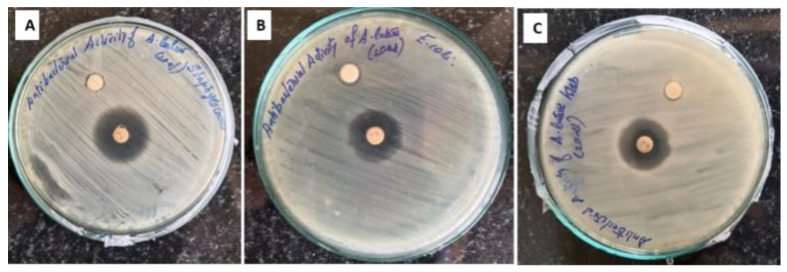
Antibacterial activity of pill millipede hemolymph against *S. aureus* (**A**) *E. coli* (**B**), and *K. pneumonia* (**C**).

The antibacterial efficacy of the hemolymph derived from the pill millipede was assessed utilizing the disc diffusion assay against three pathogenic bacterial strains: *Escherichia coli* (Migula, 1895) Castellani and Chalmers 1919, *Staphylococcus aureus* (Rosenbach, 1884), and *Klebsiella pneumonia* (Schroeter, 1886) Trevisan 1887 (Figure 6). The findings indicate that the hemolymph of the pill millipede possesses antibacterial properties, though these properties vary in effectiveness against the tested bacterial strains. The observed zones of inhibition suggest the presence of compounds within the extract capable of impeding bacterial growth. While the antibacterial activity was quantitatively less impressive when compared to commercial antibiotics, it consistently demonstrated the ability to restrict the growth of all tested pathogens. For *E. coli*, a Gram-negative bacterium, the zones of inhibition were relatively modest, signifying a lower susceptibility to the constituents of the millipede’s hemolymph. Likewise, *K. pneumonia*, also a Gram-negative bacterium, exhibited limited zones of inhibition, indicating a degree of resistance to the active compounds present in the extract. Conversely, *S. aureus*, a Gram-positive bacterium, displayed marginally larger zones of inhibition, reflecting a heightened sensitivity to the hemolymph.

### 3.6. Identification of Potential Targets

**Figure 7:**
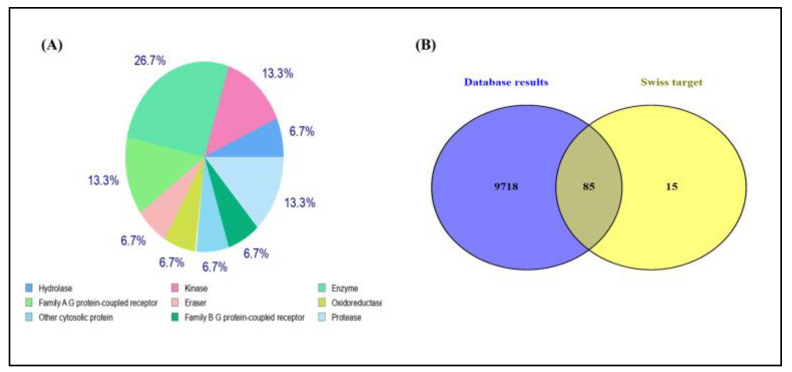
Predicted target class of DMBP using Swiss target server (**A**). Venn diagram of T-cell lymphoma targets and DMBP targets (**B**).

The Swiss target prediction web server was utilized to identify potential protein targets of DMBP, yielding approximately 101 targets. Among the top 15 predicted target classes, 26.7% were enzymes, 13.3% belonged to the kinase family, and another 13.3% were classified as family A G protein-coupled receptors. Hydrolases, oxidoreductases, proteases, and family B G protein-coupled receptors each constituted 6.7% of the targets (Figure 7A). Potential targets for Dalton’s lymphoma ascites were obtained from the Gene Cards (10,534), OMIM (145), and DisGeNET (54) databases using the keywords “Dalton’s lymphoma ascites” and “T-cell lymphoma”. A total of 9,804 gene targets were identified after removing duplicates, which were subsequently used for further analysis. Common targets from both of the sets were identified by drawing a Venn diagram, and 85 predicted targets of DMBP were suggested to have a role in T-cell lymphoma (Figure 7B).

#### 3.6.1. Construction of Protein–Protein Interaction and GO Analysis

**Figure 8:**
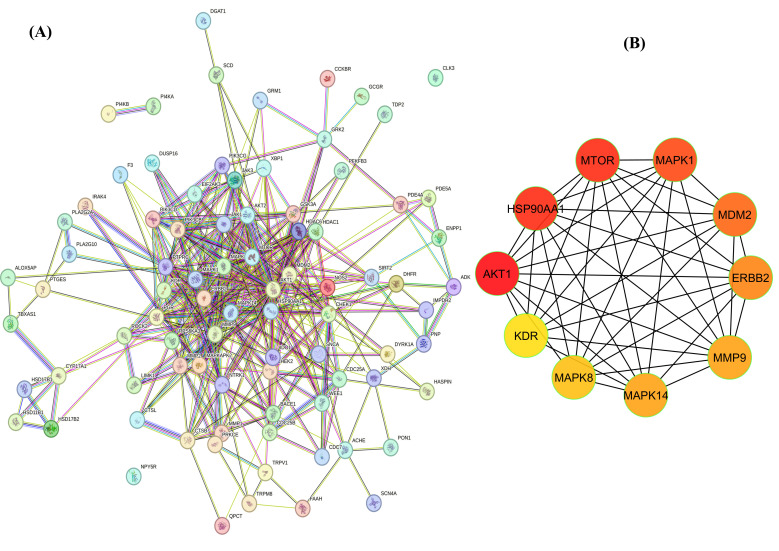
String network analysis displaying protein–protein interactions (**A**). Selection of hub genes using the Cytohubba module of Cytoscape. Color from red to yellow denotes its ranking based on its degree (**B**).

Protein–protein interaction (PPI) networks are valuable tools for understanding the roles of proteins in various biochemical processes, which are essential for comprehending cellular structure, function, and biological pathways. A PPI network involving 85 common genes was constructed using the STRING database, comprising 85 nodes and 399 edges (Figure 8A). The network was then imported into Cytoscape for further analysis. To identify hub genes, the CytoHubba plugin was employed, using the degree method to rank genes based on their connectivity. The top-ranked genes identified were AKT1, HSP90AA1, MTOR, MAPK1, MDM2, ERBB2, MMP9, MAPK14, MAPK8, and KDR, all of which exhibited significantly high-degree values (Figure 8B).

#### 3.6.2. Gene Ontology Analysis

**Figure 9:**
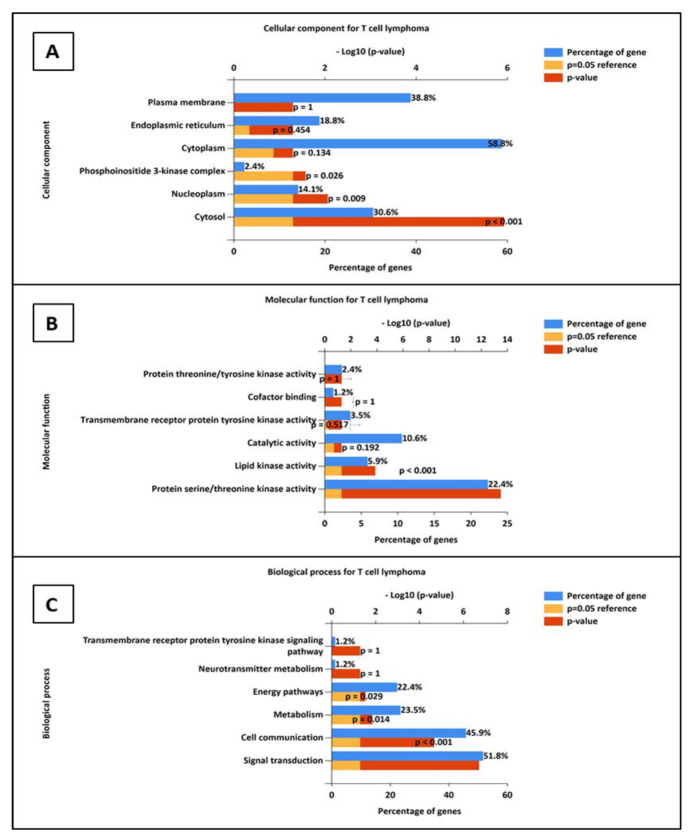
Gene ontology enriched terms: Cellular components (**A**), molecular function (**B**), and biological process (**C**).

The gene ontology (GO) analysis reveals significant enrichment of genes in key cellular components. A large proportion of the genes (30.6%) are associated with the cytosol, with a highly significant *p*-value (<0.001), suggesting active involvement in fundamental cellular processes such as protein synthesis, metabolism, and signal transduction. Additionally, 14.1% of genes are located in the nucleoplasm, with a *p*-value of 0.009, indicating significant enrichment in processes related to gene transcription, mRNA processing, and chromatin remodeling. The phosphoinositide 3-kinase (PI3K) pathway, although representing only 2.4% of the genes, is moderately enriched (*p*-value = 0.026), highlighting its role in cell survival, growth, and signaling pathways (Figure 9A).

The gene ontology (GO) analysis of the provided dataset reveals significant enrichment in specific molecular functions, highlighting key biological processes overrepresented in the gene set. Notably, protein serine/threonine kinase activity and lipid kinase activity exhibit strong enrichment, with fold enrichments of 13.47 and 30.51, respectively, and highly significant *p*-values, even after correction for multiple comparisons using methods like Bonferroni, Benjamini–Hochberg (BH), and Storey–Tibshirani Q-value. These results suggest a significant overrepresentation of these kinase activities in the dataset, which are crucial for regulating a variety of cellular processes, including signal transduction and metabolic pathways (Figure 9B).

The gene ontology (GO) analysis highlights several biological processes significantly enriched in the dataset. Signal transduction shows the highest enrichment with 51.76% of genes involved, a highly significant *p*-value (1.08 × 10^−9^), and a fold enrichment of 2.39, indicating that this process plays a central role in the dataset. Similarly, cell communication (45.88% of genes) also exhibits strong enrichment, with a significant *p*-value (1.21 × 10^−7^), suggesting its importance in cellular interactions. Metabolism and energy pathways, represented by 23.53% and 22.35% of genes, respectively, show moderate enrichment (*p*-values 7.82 × 10^−5^ and 0.00016) and are critical for energy production and cellular functioning. Neurotransmitter metabolism and transmembrane receptor protein tyrosine kinase signaling pathways, although only involving 1.18% of genes each, show very high fold enrichment (107.13) but are less significant due to high *p*-values (0.009 and 1, respectively). Overall, the dataset is enriched in processes like signal transduction, cell communication, and metabolism, indicating their key role in cellular functions (Figure 9C).

#### 3.6.3. Molecular Docking

**Figure 10:**
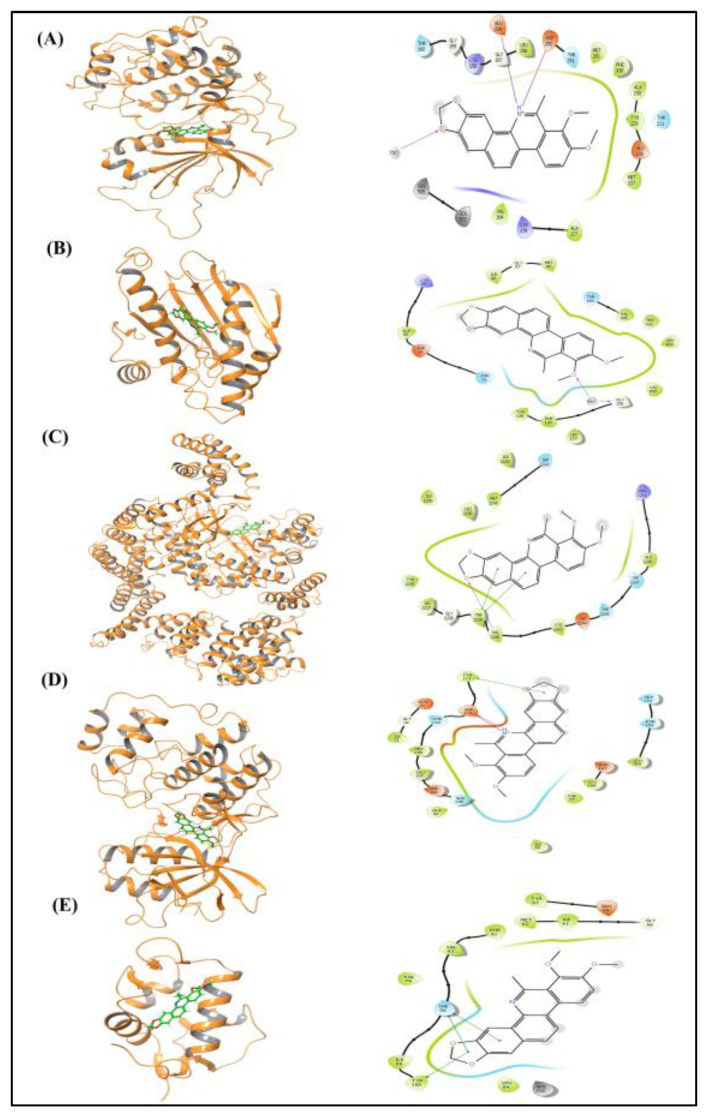
Three- and two-dimensional schematic drawings of interactions between DMBP and respective targets, AKT1 (**A**), HSP90AA1 (**B**), MTOR (**C**), MAPK1 (**D**), and MDM2 (**E**).

The docking analysis of compound DMBP against various protein targets revealed diverse binding affinities and interaction profiles (Table 4). The interactions between DMBP and its targets, including AKT1, HSP90AA1, MTOR, MAPK1, and MDM2, are illustrated in both 3D and 2D schematic drawings presented in Figure 10. For AKT1, the dock score was −2.091, with an MMGBSA value of −71.0, and key interactions included ionic bonds with Glu234 and Asp292. HSP90AA1 showed a dock score of −4.907 and an MMGBSA of −35.9; here, the interaction was mainly stabilized by van der Waals forces. MTOR exhibited a dock score of −8.5 and an MMGBSA value of −65.6, with two pi bond interactions involving Trp2239 and a hydrogen bond with Val2240. MAPK1 had a dock score of −3.529 and an MMGBSA value of −79.50, forming a pi bond with Tyr113 and interactions with Asp111, including one hydrogen bond and one salt bridge. Finally, MDM2 showed a dock score of −4.94 and an MMGBSA value of −51.13, with pi bond interactions involving Tyr100 and His 96, the latter forming two pi bonds. These results highlight a range of binding affinities and interaction types, suggesting that DMBP may exhibit varying potential for targeting these proteins.

### 3.7. Mode of Action of DMBP Against Staphylococcus aureus (Rosenbach, 1884)

**Figure 11:**
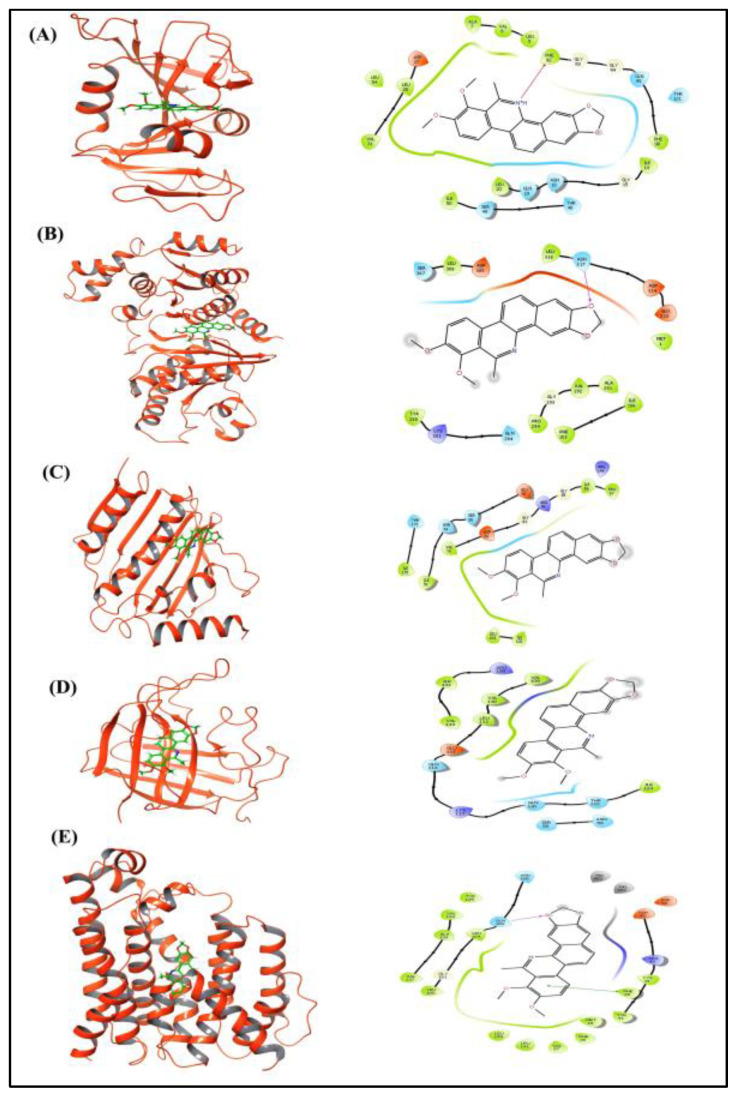
Three- and two-dimensional schematic drawings of interactions between DMBP and respective targets of *Staphylococcus aureus*: dihydrofolate reductase (**A**), phosphate acetyltransferase (**B**), DNA gyrase (**C**), sortase-A (**D**), and dehydrosqualene synthase (**E**).

Docking analysis of compound DMBP with key *Staphylococcus aureus* protein targets reveals promising interactions, highlighting its potential as a broad-spectrum inhibitor (Table 5). The 3D and 2D schematic drawings that illustrate the interactions of DMBP with key targets from *Staphylococcus aureus*, including dihydrofolate reductase, phosphate acetyltransferase, DNA gyrase, sortase-A, and dehydrosqualene synthase, are included in Figure 11. DMBP binds to dihydrofolate reductase with a docking score of −4.44 kcal/mol and a binding energy of −52.99 kcal/mol, interacting with Phe92 and potentially disrupting folate metabolism. Phosphotransacetylase shows a binding affinity of −3.396 kcal/mol and a binding energy of −47.89 kcal/mol, forming a hydrogen bond with Asn317. Strong interaction was observed with DNA gyrase, with a docking score of −5.1 kcal/mol and a binding energy of −65.3 kcal/mol, suggesting inhibition of this enzyme is critical for DNA replication and maintenance. The compound also targets sortase-A, with a docking score of −3.07 kcal/mol and a binding energy of −51.28 kcal/mol, potentially interfering with bacterial cell wall biosynthesis. Additionally, DMBP exhibits a binding affinity of −5.5 kcal/mol and a binding energy of −9.78 kcal/mol with dehydrosqualene synthase, interacting with Phe22 via a pi bond and Gln165 through a hydrogen bond, indicating potential disruption of sterol biosynthesis. These findings suggest that DMBP could effectively inhibit multiple essential enzymes in *S. aureus*, offering a potential novel therapeutic approach against this pathogen.

## 4. Discussion

Millipedes are well known for their ecological significance and their defensive property, but there is little information about the biochemical composition of their hemolymph compared to other arthropods. The hemolymph of *A. lutescens* (Butler, 1872) exhibits a sophisticated chemical strategy akin to what is observed in various arthropods. Earlier studies revealed that carbohydrates, organic acids, alcohols, lipids, organic phosphate, hydrocarbons, proteins, and amino acids are the characteristic compounds present in the hemolymph of arthropods. The GCMS analysis of the hemolymph of *A. lutescens* reveals the presence of compounds like tridecanoic acid, pentadecanoic acids, hexadecanoic acids, heptadecanoic acids, octadecanoic acid, neophytadiene, and esters like methyl ester and alcohols like 9-octadecen-1-ol and cholesterol. The most abundant compound from the analysis is fatty acids, which are commonly found in the hemolymph of arthropods. Hexadecanoic acid, pentadecanoic acid, and neophyatadiene are documented in the hemolymph of the scarabid beetle *Scarabaeus sacer* (Linnaeus, 1758), and palmitic acid and oleic acid are reported in the hemolymph of *Spodoptera littoralis* (Boisduval, 1833) [16,41]. The presence of docosanoic acid, pentadecanoic acid, and octadecanoic acid was observed in the GC-MS analysis of the hemolymph of the giant African snail *Arachatina marginata* (Swainson, 1821) [42]. Some of the components obtained from the hemolymph of *A.lutescens* also have a significant role in the chemical secretion of millipedes; e.g., a carboxylic acid present in the pygidial gland acts as a deterrent against predators [43]. Hexadecanoic acid is a usual chemical component present in insect secretions, which acts as a precursor to the production of longer fatty acid chains [44]. Further, it is involved in the metabolic process.

Several compounds identified through GC-MS analysis were found in Dr. Duke’s phytochemical and ethnobotanical databases [36]. These include neophytadiene, which possesses analgesic, antipyretic, anti-inflammatory, and antimicrobial properties. 9-Octadecenoic acid is well known for its anti-inflammatory, anti-alopecic, anemiagenic, 5-reductase inhibitor, α-reductase inhibitor, lubricant, antitumor, choleretic, dermatitigenic, immunostimulant, anti-leukotriene-D4, antiandrogenic, lipoxygenase inhibitor, allergenic, flavor, hypocholesterolemic, insectifuge, irritant, percutaneous stimulant, and propecic properties. Hexadecanoic acid is another commonly identified compound with lubricant, antiandrogenic, antioxidant, and 5-alpha-reductase inhibitor properties. Pentadecanoic acid is recognized for its antioxidant properties, while methyl ester demonstrates both antioxidant and anti-inflammatory effects.

Sugars play a key role in providing an energy source for insect metabolism; furthermore, they also they protect insects from stress conditions by optimizing proteins in the cells [45]. Trehalose is the major sugar found in arthropod hemolymph [46]. Glucose, trehalose, turanose, lactose, mannobiose, maltose, inulobiose, and gentiobiose are the prominent sugars observed in the hemolymph of *A. lutescens*. More than 75 carboxylic acids are determined through LC-MS analysis; most of them are common in the hemolymph of arthropods. Fatty acid esters like butyl dodecanote, pentyl decanote, methyl tetradecanote, 3 methyl butyl dodecanate, ethyl decanote, and butyl dodecanate are the abundant esters reported in the hemolymph of *A. lutescens.*

The amino acids present in the insect hemolymph have a crucial role in osmoregulation, protein synthesis, and energy storage [47]. The reported amino acids in the LC-MS analysis of the hemolymph of pill millipedes are phenylalanine, isoleucine, proline, serine, tryptophan, tyrosine, and valine. These amino acids are also reported in the hemolymph of the land snail *Helix pomatia* (Linnaeus, 1758) [48]. 4-[1-ethyl-2-(4- fluorophenyl) butyl] phenol, 2-butyl-4-methylphenol, 4-isopentylphenol, n-pentylphenol, and naphthalene dihydrodiol are the major phenolic compounds discovered by LC-MS analysis of *A. lutescens* secretion. 1,2-dimethoxy-13-methyl-[1,3]benzodioxolo[5,6-c] phenanthridine is the abundant alkaloid observed in the hemolymph of *A. lutescens,* which is also found in Zanthoxylum species. Zanthoxylum is the most common plant that we observed in the Seetharkundu region. Valerianine is another alkaloid observed in valerian plants and is well known for its sedative and anxiolytic effect [49]. 2-(3-Phenylpropyl) pyridine is an alkaloid with antimicrobial, antifungal, and anti-inflammatory biological activities also observed in the hemolymph of *A. lutescens*. Among the identified fatty acids, myristic acid is a saturated fatty acid ester found in the defensive secretion of millipedes, e.g., *Pseudopolydesmus serratus* (Say, 1821) [50]. Benzoic acid is an aromatic carboxylic acid commonly documented in the defensive secretion of the millipedes *Gomphodesmus pavani* (Demange, 1965), *Orthomorpha coarctata* (De Saussure, 1860)*,* and *Polydesmus collaris* (Latzel, 1884) [51]. This carboxylic acid exhibits significant antimicrobial activity [52]. Tetradecanoic acid is a significant fatty acid documented in the phasmid *Eurycantha calcarata* (Lucas 1869) also observed in the LC-MS of *A. lutescens* with potential antimicrobial activity against fungi and potential anticancer activity. Myristic and palmitic acids are known for their antibacterial activity and repellent against predators [53]. The diverse hemolymph chemical repertoire exemplifies a comprehensive strategy employed by *A. lutescens* in response to various biological processes.

The DPPH radical scavenging activity exhibited by the hemolymph of pill millipedes reflects its potential as a natural antioxidant. The findings of this study are further substantiated by extensive entomological research, which frequently reveals significant antioxidant properties in arthropod hemolymph. For instance, insects such as diptera have demonstrated comparable DPPH scavenging capabilities, indicating a shared evolutionary strategy among arthropods to combat oxidative stress [54]. The specific chemical constituents identified, including various phenolic and terpenoid compounds, are well recognized for their electron donation abilities, which are vital for neutralizing free radicals [55]. Of particular note is the compound 1,2-dimethoxy-13-methyl-[1,3]benzodioxolo[5,6-c]phenanthridine. This phenanthridine derivative is esteemed for its potent antioxidant properties, attributed to the presence of aromatic rings that facilitate electron donation. This structural feature is instrumental in neutralizing free radicals, contributing to the overall DPPH radical scavenging activity.

The antibacterial activity exhibited against *Escherichia coli* (Migula, 1895) Castellani and Chalmers 1919 and *Staphylococcus aureus* (Rosenbach, 1884) is moderate yet significant, indicating the presence of broad-spectrum antibacterial agents within the hemolymph of the pill millipede. Such findings are consistent with research conducted on other arthropods, notably the studies regarding the hemolymph of beetles, which have identified specific alkaloids and peptides exhibiting both bacteriostatic and bactericidal properties [16]. The mechanisms of action may involve the disruption of bacterial membranes or inhibiting essential bacterial enzymes, a common feature among natural antibacterial compounds [56]. Specifically, the hemolymph demonstrated mild antibacterial efficacy. Naphthalene di-hydro diol has been recognized in several instances and is documented in the literature for its antimicrobial properties, likely due to its ability to interfere with bacterial cell wall synthesis or disrupt membrane integrity. The quinones found in the hemolymph are likely significant contributors to its antimicrobial properties. Furthermore, the compound 3b-hydroxy-6b-(3-chloro-2-hydroxy-2-methylbutanoyloxy)-7(11)-eremophilen-12,8b-olide possesses a complex structure inclusive of a chloro group that may enhance its antibacterial effectiveness, potentially through mechanisms that disrupt microbial membrane structures or metabolic pathways.

The observed anticancer activity against Dalton’s lymphoma ascites (DLA) cells indicates that certain compounds present in the millipede’s hemolymph can induce cytotoxic effects in cancerous cells. Notably, the compound 1,2-dimethoxy-13-methyl-[1,3]benzodioxolo[5,6-c]phenanthridine, identified in this study, demonstrates significant promise due to its prevalence and preliminary bioactivity profile. The structural characteristics of this compound, particularly the benzodioxolo moiety, are expected to interfere with DNA replication or repair mechanisms in cancer cells. This hypothesis aligns with proposed modes of action for similar compounds found in nature [57]. The phenanthridine derivative is believed to play a crucial role in this activity through its interaction with DNA, potentially inhibiting nucleic acid synthesis and inducing apoptosis in malignant cells. The distinctive structure of this phenanthridine enhances its ability to bind to and disrupt DNA, indicating its potential as a promising lead compound in the development of anticancer therapeutics [58].

To explore the possible protein targets of DMBP, we used the Swiss target prediction web server, which identified around 101 predicted targets. Enzymes represented the largest category among the top 15 target classes, followed by kinases and family A G protein-coupled receptors (GPCRs). Additional target classes included hydrolases, oxidoreductases, proteases, and family B GPCRs. To evaluate the potential significance of these targets in relation to Dalton’s lymphoma ascites, disease-associated genes were gathered from key databases—GeneCards (10,534 genes), OMIM (145 genes), and DisGeNET (54 genes). We conducted a comparative analysis using a Venn diagram to identify shared targets between the DMBP-predicted proteins and the disease-associated genes. This analysis uncovered 85 common targets, indicating that DMBP may hold therapeutic potential in T-cell lymphoma by influencing genes involved in the disease’s pathogenesis.

Protein–protein interaction (PPI) network analysis provides a systems-level perspective on how proteins function in concert to regulate cellular processes [59]. In the present study, a PPI network was constructed using 85 genes common to both the predicted targets of DMBP and T-cell-lymphoma-associated genes. Subsequent analysis using Cytoscape and the CytoHubba plugin, based on the degree of connectivity, enabled the identification of key hub genes within the network. The top-ranking hub genes—AKT1, HSP90AA1, MTOR, MAPK1, MDM2, ERBB2, MMP9, MAPK14, MAPK8, and KDR—exhibited high degrees of interaction, suggesting their central roles in maintaining the structural integrity and functional coherence of the network. Many of these genes are well-established regulators of cell growth, proliferation, apoptosis, and signal transduction [60]. For instance, AKT1 and MTOR are core components of the PI3K/AKT/mTOR pathway, a crucial axis in cancer cell survival and metabolism [61]. The identification of MDM2, a negative regulator of p53, further supports a potential role for DMBP in modulating apoptotic pathways [62]. HSP90AA1 and ERBB2, known for their roles in protein folding and receptor tyrosine kinase signaling, respectively, highlight DMBP’s potential to interfere with oncogenic signaling DMBP targets but also identify key regulatory nodes that may serve as critical mediators of its therapeutic activity [63]. These hub genes represent promising candidates for further validation and functional investigation to elucidate the molecular mechanisms underlying DMBP’s potential anti-lymphoma effects.

The molecular docking and MMGBSA analysis of the compound DMBP against a panel of lymphoma-associated protein targets—AKT1, HSP90AA1, MTOR, MAPK1, and MDM2—revealed distinct binding affinities and interaction profiles, indicative of its potential as a multi-target therapeutic agent. Among the evaluated targets, DMBP exhibited a particularly favorable interaction with MTOR, primarily mediated by aromatic stacking and hydrogen bonding. The presence of these non-covalent interactions suggests that DMBP is capable of adopting a stable and energetically favorable conformation within the MTOR binding pocket. Given MTOR’s central role in regulating cell proliferation and survival, such an interaction profile supports the potential utility of DMBP in modulating this critical signaling pathway in lymphoma.

The molecular docking and binding energy analysis of DMBP against essential *Staphylococcus aureus* (Rosenbach, 1884) enzymes reveal a multi-target inhibitory potential, suggesting a promising mode of action for this compound as an antimicrobial agent. DMBP exhibited notable binding affinity towards dihydrofolate reductase (DHFR), interacting with active site residue Phe92. As DHFR plays a central role in folate metabolism and nucleotide biosynthesis [64], this interaction suggests that DMBP may hinder DNA and RNA synthesis, thereby impairing bacterial replication. A particularly strong interaction was observed with DNA gyrase, a well-established antibacterial target responsible for DNA supercoiling and replication [65]. The significant binding energy suggests that DMBP may act as a potent inhibitor of DNA gyrase, potentially leading to replication arrest and cell death. DMBP interacted effectively with dehydrosqualene synthase, a key enzyme in the biosynthesis of staphyloxanthin, a virulence-associated carotenoid [66]. The interaction, involving both hydrogen bonding and aromatic stacking, suggests a capacity to inhibit pigment synthesis, potentially attenuating bacterial resistance to oxidative stress.

## 5. Conclusions

This investigation highlights the significant pharmaceutical potential of metabolites extracted from the hemolymph of the giant pill millipede A. lutescens (Butler, 1872). We have mapped a complex chemical landscape within the hemolymph. Notably, the hemolymph demonstrated significant radical scavenging, antibacterial, and cytotoxic activities, underlining its potential as a source of new therapeutic agents. The metabolite 1,2-dimethoxy-13-methyl-[1,3]benzodioxolo[5,6-c]phenanthridine (DMBP), in particular, has shown promising results in both antibacterial efficacy against S. aureus and cytotoxicity against lymphoma cells, as further supported by molecular docking studies targeting key proteins involved in T-cell lymphoma and bacterial processes. These findings not only enhance our understanding of the chemical diversity in the hemolymph of pill millipedes but also set a foundation for future pharmacological explorations, potentially leading to innovative treatments for both infectious and oncological diseases.

## Figures and Tables

**Table 1:** Bioactive components identified in the hemolymph of the giant pill millipede *A. lutescens*.

Compound Name	Molecular Formula	Nature of Compound	Retention Time	Peak Area %
Methyl tetradecanoate	C_15_H_30_O_2_	Ester	17.195	0.48
9-Octadecen-1-ol	C_18_H_36_O	Alcohol	17.280	0.24
Tridecanoic acid	C_17_H_34_O_2_	Fatty acid	17.723	0.59
Pentadecanoic acid, methyl ester	C_16_H_32_O_2_	Fatty acid	17.892	1.76
Neophytadiene	C_20_H_38_	Alkene	18.429	0.42
1,2-Benzenedicarboxylic acid	C_16_H_22_O_4_	Carboxylic acid	18.694	0.60
Hexadecanoic acid	C_17_H_34_O_2_	Fatty acids	18.955	1.00
(Z)-Methyl hexadec-11-enoate	C_17_H_32_O_2_	Ester	19.221	2.17
Tridecanoic acid, 4,8,12-trimethyl-, methyl ester	C_17_H_34_O_2_	Fatty acids, ester	19.814	0.31
Heptadecanoic acid, methyl ester	C_18_H_36_O_2_	Fatty acids, ester	19.968	1.32
cis-10-Heptadecanoic acid, methyl ester	C_18_H_34_O_2_	Fatty acids, ester	20.104	0.55
9,12-Octadecadienoic acid (Z, Z)-, methyl ester	C_19_H_34_O_2_	Fatty acids, ester	20.988	9.09
9-Octadecenoic acid, methyl ester	C_19_H_36_O_2_	Fatty acids, ester	21.049	18.06
11-Octadecenoic acid, methyl ester	C_19_H_36_O_2_	Fatty acids, ester	21.099	3
Methyl stearate	C_19_H_38_O_2_	Fatty acid ester	21.283	2.72
Eicosatetraenoic acid, methyl ester	C_21_H_34_O_2_	Fatty acids, ester	22.488	1.11
Docosenoic acid, methyl ester	C_23_H_44_O_2_	Fatty acids, ester	24.493	1.71
Cholesterol	C_27_H_46_O	Alcohol	30.577	35.68

**Table 2:** Activities of some selected compounds identified in the hemolymph of the giant pill millipede *A. lutescens*.

Compound Name	Bioactivity
Methyl tetradecanoate	Anticancer activity [33]
9-Octadecen-1-ol	Non-ionic surfactant, thickener, and emulsifier [34]
Tridecanoic acid	Antibacterial activity [35]
Pentadecanoic acid	Antioxidant properties [36]
Neophytadiene	Analgesic, antipyretic, anti-inflammatory, and antimicrobial properties [36]
1,2-Benzenedicarboxylic acid	Cytotoxic activity [37] and anti-inflammatory activity [38]
Hexadecanoic acid	Antioxidant, anti-inflammatory, and antibacterial activity [39]
9-Octadecenoic acid	Anticancer activity and antioxidant activity [40]
Methyl stearate	Antioxidant and anti-inflammatory effects [36]

**Table 3:** Metabolites identified in the hemolymph of *A. lutescens* by LC-MS analysis.

Class of Compounds	Compound Name	Molecular Formula	Retention Time	Mass-to-Charge Ratio	Scores
Sugars	Trehalose	C_12_H_22_O_11_	1.529	342.1156	96.38
	Lactose	C_12_H_22_O_11_	1.529	342.1156	96.38
	Mannobiose	C_12_H_22_O_11_	1.529	342.1156	96.38
	Maltose	C_12_H_22_O_11_	1.529	342.1156	96.38
	Inulobiose	C_12_H_22_O_11_	1.529	342.1156	96.38
	Gentiobiose	C_12_H_22_O_11_	1.529	342.1156	96.38
Amino acids/modified amino acids	Phenylalanine	C_9_H_11_NO_2_	3.539	166.08	94.29
	N, N-Diethylglycine	C_6_H_13_NO_2_	2.193	131.0937	94.21
	Isoleucin	C_6_H_13_NO_2_	2.193	131.0937	94.21
	Leucine	C_6_H_13_NO_2_	2.193	131.0937	94.21
	Anaserine	C_10_H_16_N_4_O_3_	31.551	240.1220	93.667
	Na-Hexanoyl-Nb-inosityltryptophan	C_23_H_32_N_2_O_8_	23.244	464.2135	93.59
	Phenylalanyl-Tyrosine	C_18_H_20_N_2_O_4_	10.666	328.143	90.11
	Valine	C_5_H_11_NO_2_	1.529	117.0786	90.29
Fatty acids	Methyl myristic acid	C_15_H_30_O_2_	38.097	242.2244	99
	Tetradecanoic acid	C_14_H_28_O_3_	30.620	244.2028	95.33
	Tridecanoic acid	C_13_H_26_O_2_	33.063	214.1926	97.26
	Palmitic acid	C_16_H_32_O_2_	34.907	256.2400	96.61
	Lauric acid	C_12_H_24_O_2_	30.521	200.1767	96.38
Esters	Butyl dodecanote	C_16_H_32_O_2_	34.907	256.2400	96.61
	Pentyl decanoate	C_15_H_30_O_2_	38.097	242.2244	99
	Methyl dodecanote	C_13_H_26_O_2_	33.063	214.1926	97.86
	Ethyl decanoate	C_12_H_24_O_2_	30.521	200.1767	96.38
Alkaloids	1,2-Dimethoxy-13-methyl-[1,3]benzodioxolo[5,6-c]phenanthridine	C_21_H_17_NO_4_	19.107	347.1164	92.116
	Valerianine	C_11_H_15_NO	35.056	177.1152	97.79
	2-(3Phenylpropyl) pyridine	C_14_H_15_N	18.475	197.1196	95.03
Phenols	4-(1-Ethyl-2-ph enyl butyl) phenol	C_18_H_22_O	28.843	254.1664	92.63
	4-Isopentylphenol	C_11_H_16_O	28.643	164.7192	92.50
	4-n-Pentylphenol	C_11_H_16_O	28.643	164.1192	92.50
	Naphthalene dihydro diol	C_10_H_10_O_2_	38.579	162.0678	98.65

**Table 4:** Binding affinity of various lymphoma drug targets with DMBP from molecular docking and MMGBSA analysis.

Target Name	Docking Score (kcal/mol)	MMGBSA (kcal/mol)
AKT1	−2.091	−71.0
HSP90AA1	−4.907	−35.9
MTOR	−8.5	−65.6
MAPK1	−3.529	−79.50
MDM2	−4.94	−51.13

**Table 5:** Binding affinity of various *S. aureus* drug targets with DMBP from molecular docking and MMGBSA analysis.

Target Protein	Dock Score (kcal/mol)	MMGBSA(kcal/mol)
Dihydrofolate reductase	−4.44	−52.99
Phosphate acetyltransferase	−3.396	−47.89
DNA gyrase	−5.1	−65.3
*Sortase*-*A*	−3.07	−51.27
Dehydrosqualene synthase	−5.5	−9.78

## Data Availability

The data generated and analyzed during the current study are available from the corresponding author upon reasonable request.
